# Mitral stenosis in a teenager after rheumatic mitral valve regurgitation valve repair: A case report

**DOI:** 10.3389/fcvm.2022.978874

**Published:** 2022-12-15

**Authors:** Neguemadji Ngardig Ngaba, Uzoego Nwakaku Chibuzo, Meet Patel, Amit Gulati, Olatunde Ola, Allarangué Djindimadje, Imteyaz A. Khan

**Affiliations:** ^1^CHU Bon Samaritain de Walia, N’Djamena, Chad; ^2^Department of Pediatrics, Jamaica Hospital Medical Center, New York, NY, United States; ^3^Interfaith Medical Center, Brooklyn, NY, United States; ^4^Maimonides Medical Center, Brooklyn, NY, United States; ^5^Division of Hospital Medicine, Mayo Clinic Graduate School of Biomedical Sciences, Mayo Clinic Health System, La Crosse, Wisconsin and Center for Clinical and Translational Science, Rochester, MN, United States; ^6^Rutgers Robert Wood Johnson Medical School, New Brunswick, NJ, United States

**Keywords:** rheumatic mitral regurgitation, mitral valve (MV) repair, complication, mitral stenosis, case report

## Abstract

**Introduction:**

Mitral stenosis (MS) is a widely known complication of mitral valve repair for non-rheumatic mitral regurgitation (MR). Few reports are available on the occurrence of MS after mitral valve repair for rheumatic MR in young populations.

**Case summary:**

A 14-year-old girl presented with orthopnea, abdominal distension, and bilateral lower-limb edema. She was cachectic, with a high-pitched holosystolic murmur best heard at the cardiac apex, bilateral basal crackles, tender hepatomegaly, pitting pedal edema, and jugular venous distension. Antistreptolysin O (ASO) titer was elevated. Transthoracic echocardiography (TTE) revealed the loss of central coaptation of the mitral valve with leaflet restriction and MR, annular dilatation of the tricuspid valve, and tricuspid regurgitation (TR). She had AHA/ACC stage D mitral and TR s. Tricuspid annuloplasty and mitral valve repair for rheumatic MR were performed using Carpentier Edwards numbers 30 and 34, respectively. Following surgery, the weight and body mass index (BMI) rapidly normalized. The patient also developed progressive MS.

**Discussion:**

Previous studies in adults have described the etiopathogenesis of MS after non-rheumatic mitral valve repair. There is a paucity of reports describing the development of MS over the span of months after rheumatic MR valve repair in early pubescent children.

**Conclusion:**

Growth spurts during puberty can potentially affect MR repair, as the mitral valve prosthesis based on the preoperative Body Surface Area (BSA) is outgrown. There is a need for research on planning, prognostication, and development of an optimal, individualized, and adaptable approach to MR intervention in early pubescence.

## Introduction

The first successful mitral valve repair was performed for rheumatic mitral stenosis (MS) by Dr. Elliot Cutler at Brigham and Women’s Hospital of Boston in 1923 (1).

It was Dr. C. Walton Lillehei from the University of Minnesota who did the first mitral valve repair for mitral regurgitation (MR) by annuloplasty in 1957 (2).

The implementation of mitral valve repair of MR beyond the degenerative etiology (3, 4) has spread because of the meaningful contributions of Carpentier (5), Duran et al. (6), and many cardiac surgeons (7, 8).

Recent studies (9–12) have shown a relative benefit of mitral valve repair for organic MR over mitral valve replacement. Despite the improvements and advances in the procedure, there are still some complications after mitral valve repair.

MS is a known complication after surgical mitral valve repair for MR (13).

Currently, functional MS is defined as a mean transmitral pressure gradient (TMPG) of > 5 mmHg or mitral valve area (MVA) of < 1.5 cm^2^ regardless of the cause (14, 15).

The development of MS after mitral valve repair has not been well investigated despite its high incidence ranging from 9 to 54% (15–17).

Several studies (18–20) have reported the development of MS after non-rheumatic MR valve repair in adult patients.

The literature has not yet described the cases of MS after mitral valve repair using the Carpentier Edwards ring for rheumatic MR in the younger population or the mechanism behind the occurrence of this complication.

Herein, we report the case of a 14-year-old girl who developed progressive severe MS after valve mitral repair using Carpentier Edwards ring 34 for severe rheumatic MR. We recapped the clinical and paraclinical aspects of the patient in preoperative status and postoperative (POD) follow-up for up to many years. Knowing that the peak height velocity of 8–10 cm per year (21, 22) occurs prior to the onset of menses, and according to the five Tanner stages (23) of puberty, this study chronologically correlated patient development with POD clinical findings. The study also reported the intraoperative mechanism of the affected cardiac valves and the repair surgical technique was performed accordingly.

## Case description

A 14-year-old female patient presented with an 8-month history of new-onset murmur, progressively worsening palpitations, orthopnea, chest pain, and syncope. She was 158 cm tall, weighed 37 kg, and was cachectic with a BMI at the first centile for age and sex (14.8 kg/m^2^). Apart from tachycardia, vital signs were normal for age and sex (blood pressure 110/70 mm Hg; temperature 36.5°C; heart rate 110 beats/min). Tanner stage was 2 (downy pubic hair and palpable breast tissue without areola enlargement).

Physical examination findings were as follows: 4/6 MR murmur (24, 25) and regular tachycardia at 110/min; bilateral pulmonary crackles with dullness to percussion in the lower lobes; jugular venous distention and tender hepatomegaly with a liver span of 18 cm; and ascites and mild bilateral lower extremity edema classified as grade 2 + (26).

Antistreptolysin O (ASO) titers were elevated. Chest radiography and electrocardiography revealed a cardiothoracic ratio of 0.6 and left atrial enlargement with a cardiac axis of 30°.

Transthoracic echocardiography (TTE) revealed a mitral valve annulus diameter of 27 mm, severe MR due to leaflet restriction and loss of central coaptation, proximal isovelocity surface area (PISA) radius measuring 10 mmHg, and a mean gradient of 100 mmHg, an anatomically normal tricuspid valve with a hiatus measuring approximately 3 mm and an axial leak reaching the bottom of the right atrium (RA), and elevated pulmonary artery systolic pressure (PASP) at 55 mmHg. These clinical and TTE findings classified our patient as stage D for both mitral and TR (27).

The case was addressed to the cardiac surgery team of the Hôpital Européen George Pompidou in France through the non-governmental organization named chaîne de l’Espoir.

The patient received captopril, digoxin, furosemide, aspirin, spironolactone, and benzathine penicillin while awaiting surgery.

She underwent open cardiac surgery on 2 April 2010 at the Hôpital Européen George Pompidou, Paris, France.

Intraoperative findings during cardiac surgery were as follows: posterior mitral leaflet retraction and dilatation of the tricuspid annulus; mitral leaflets were enlarged using an autologous pericardial patch for the posterior and an oval pericardial patch for the anterior leaflets; mitral and tricuspid annuli were remodeled using Carpentier-Edwards Physio annuloplasty ring numbers 34 and 30, respectively.

There were no perioperative complications, and the immediate POD period was uneventful.

**FIGURE 1:**
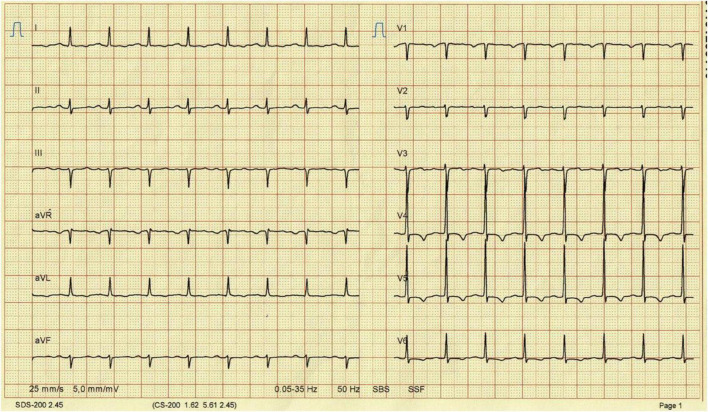
Electrocardiography reveals an atrioventricular type 1.

Clinical and paraclinical assessments were performed, which revealed tachycardia and decreased bilateral lower lung vesicular murmur. Chest radiography confirmed mild bilateral pleural effusion. Type 1 atrioventricular block was observed (Figure 1).

A serial TMPG measured in the immediate POD period revealed the following: 9 mmHg on the 4th postoperative (POD) day which was related to the tachycardia at 120 beats/min because the MVA was at 3.6 cm^2^; 2.7 mmHg on the 11th POD; and 4 mmHg on the 33rd POD.

**FIGURE 2:**
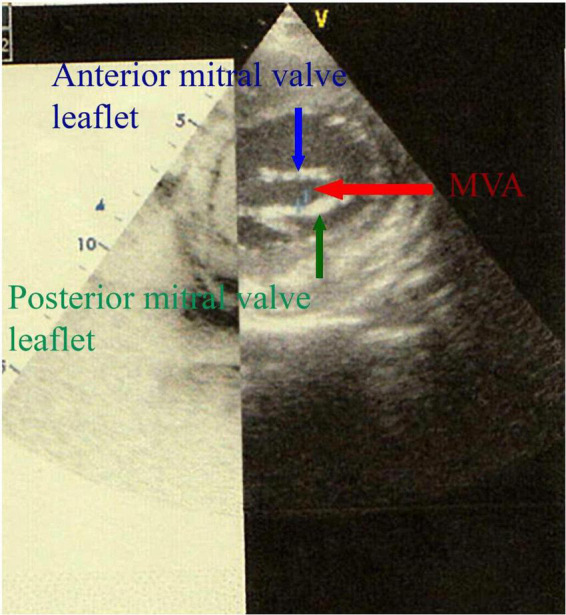
Two-dimensional (2D) transthoracic echocardiography in parasternal short-axis view shows the MVA measurement by planimetry.

**FIGURE 3:**
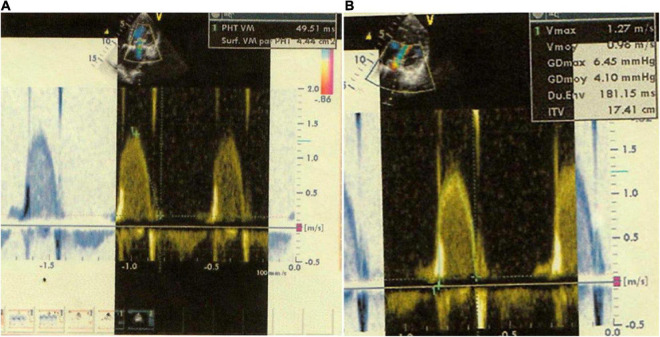
**(A)** Two-dimensional (2D) apical 4-chamber view shows a mitral valve pressure half-time (PHT) of 49.51 ms. **(B)** Two-dimensional (2D) apical 4-chamber view shows a transmitral gradient pressure of 4.10 mmHg.

A TTE was performed on the 33rd POD, which revealed a residual MR type A with an MVA of 4 cm^2^ (Figure 2), the pressure half-time (PHT) of the mitral valve at 49.51 ms (Figure 3A) with a TMPG of 4 mmHg (Figure 3B) and the left auricle at 29 cm^2^; and a residual TR type A with a PASP at 40 mmHg. None of the POD TTE (immediate, midterm, or long-term follow-up) revealed pannus formation on fibrous tissue growth of the mitral valve.

The patient was advised to follow up at the ambulatory care setting every month with a trained general practitioner to manage POD cardiac patients. At this visit, functional signs and complaints of the patient are collected, and a physical examination is performed after taking the patient’s vitals (weight, height, blood pressure, heart rate, respiratory rate, and temperature). During POD surgery, she was administered daily oral medications (furosemide, spironolactone, and acetylsalicylic acid) and monthly intramuscular penicillin benzathine. The patient has an appointment every 3–4 months with the cardiologist. At that visit, an electrocardiogram and an echocardiogram are performed as well as laboratory tests (complete blood counts, comprehensive metabolic panels, and isolated potassium). The patient was counseled about strict compliance with the medical follow-up and to come to the emergency room if any other concerns. She gradually gained weight and increased in height. Menarche occurred 17 months (October 2011) after the procedure. POD development of MS was observed by TTE, with worsening clinical findings during follow-up (Table 1).

**TABLE 1:** Postoperative follow-up findings.

Postoperative time	Distance of dyspnea on exertion	Physical examination					TTE findings			
		Weight (kg)	Height (cm)	BMI (kg/m^2^) <*percentile*>	BSA (m^2^)	Murmur	Mitral valve		TMPG (mmHg)	PASP (mmHg)
							Anatomy	Area (cm^2^)		
1 month	Not assessed	36.5	158	14.62 < 1 >	1.40	No murmur	Repaired defect	4	4	30
10 months	1 km	46	165	16.89 <1 >	1.55	Diastolic rumble	Thickened posterior leaflet	1.2	15	44
23 months	100 m	51	171	17.44 <1 >	1.62	Diastolic rumble with presystolic accentuation	Thickened posterior leaflet	1.16	16	40
34 months	100 m	59	175	19.3 <27th >	1.72	Diastolic rumble with presystolic accentuation, opening snap	Thickened posterior leaflet	1	18.7	47

BMI, body mass index; BSA, body surface area; PASP, pulmonary artery systolic pressure; TMPG, transmitral pressure gradient; TTE, transthoracic echocardiography.

## Discussion

The patient underwent rheumatic mitral valve repair for MR with a Carpentier Edwards ring, which has been known to be a good therapeutic option (9–12).

This study revealed the development of MS after MR repair in a young patient. This complication has been reported solely in the adult population undergoing non-rheumatic MR repair (18–20).

Various mechanisms (28–30) have been implicated in the development of MS after MR repair, including pannus formation, annuloplasty ring size, and subvalvular tethering.

Mitral annulus pannus formation is the predominant POD pathology in delayed MS following MR repair. Our patient underwent rheumatic mitral valve repair for MR with a rigid CE ring. Compared to flexible Duran, this therapeutic option is less commonly associated with POD MS (31). Retrospective studies (31, 32) that compared the outcomes of surgeries in patients who used CE to those who used more flexible annuloplasty options, such as the Duran ring, found a progressive reduction in POD MVA and an increase in TMPG, which were significantly more prevalent in surgeries involving the flexible annuloplasty ring. In these patients, imaging revealed pannus formation around the mitral annulus, which was implicated in the causation of their MS. However, this was not the cause of the changes in MVA and TMPG observed in our patient, as no pannus formation was observed in serial TTE.

The choice of annuloplasty ring (33) has been described as the main mechanism related to the development of early stenosis. When this is the operative mechanism, the POD TMPG is higher than the pre-operative values and the MVA is < 1.5 cm^2^. However, in our patient, both the post-operative MVA of 3.6 cm^2^ and TMPG of < 5 mmHg correlate with an appropriately sized annuloplasty ring.

Subvalvular tethering, the cause of MS in patients with ischemic MR (30), was not observed in our patient.

We hypothesized that hemodynamic changes due to rapid body growth lead to a greater BSA, higher circulating blood volume, transvalvular flow, trans-prosthetic pressures, and a decreased effective orifice area indexed to BSA (EOA). Our patient had two pre-operative characteristics that accounted for the rapid increase in the BSA after surgery. Our pre-menarchal patient did not attain peak height velocity and had a BMI below the 3rd percentile. In the first year after surgery, she attained Tanner stage 3 and experienced a corresponding peak growth velocity of 8.4 cm per year. In addition, she had a rapid increase in BMI from the 1st to 10th percentile over a period of 10 months. This rapid increase in height and weight culminated in a corresponding change in BSA, circulating blood volume, and cardiac output. However, the prosthetic ring size remained unchanged. For the transvalvular pressures to remain low, the EOA and flow requirements must be proportionate (34). As the patient size continued to increase, the EOA of the mitral valve became increasingly smaller with the BSA and cardiac output leading to a prosthesis patient mismatch. We hypothesized that this was due to the observed elevation in TMPG.

Different mechanisms have been reported to lead to the occurrence of early or delayed MS after MV repair, which includes surgical technique (16), elevated mean TMPG (15, 35), annuloplasty that reduces ring size (30), and pannus formation on fibrous tissue growth (36). The increase in cardiac output in our patient, proportional to the considerable weight and height gain and the lack of mitral annulus area growth, can account for the development of progressive MS, which is similar to a mismatch between cardiac output and MVA.

Our case is just to reveal the occurrence of this complication in a growing young population, which was added to previous in-depth studies to explain this phenomenon.

## Conclusion

Growth spurts in pubescent children have hemodynamic effects that can potentially affect the outcome of MR repair, as children outgrow a mitral valve prosthesis matched based on a projected EOA at their preoperative BSA. Identification of similar groups of patients at risk for post-reconstruction sequela due to hemodynamic changes that occur with rapid growth may assist in prognostication and planning prior to mitral valve surgery. This case also demonstrates the need for further research on an optimal, individualized, and adaptable approach to MR interventions in early pubescence.

## Ethics statement

The studies involving human participants were reviewed and approved by the Bioethics Committee of Chad. The patients/participants provided their written informed consent to participate in this study. Written informed consent was obtained from the patient for publication of any potentially identifiable images or data included in this case report.

## Author contributions

NN, UNC, MP, AG, OO, AD, and IAK: case treatment, manuscript preparation, diagnosis, and literature review. All authors contributed to the study and approved the submitted manuscript.
